# Bullous Type 2 Lepra Reaction: A Rare and Significant Case Report

**DOI:** 10.1002/ccr3.70056

**Published:** 2025-01-06

**Authors:** Hrithik Dakssesh Putta Nagarajan, Balakrishnan Kamaraj, Keerthivasan Selvanathan, Pruthvi Raj Kukunoor, Katta Manichandana, Katta Manideep, Biki Kumar Sah

**Affiliations:** ^1^ Department of Internal Medicine Madurai Medical College Madurai Tamil Nadu India; ^2^ Department of Dermatology Madurai Medical College Madurai Tamil Nadu India; ^3^ Department of Internal Medicine Osmania Medical College Hyderabad Telangana India; ^4^ Department of Internal Medicine B.P. Koirala Institute of Health Sciences Dharan Nepal

**Keywords:** communicable diseases, dermatology, internal medicine, leprosy

## Abstract

Bullous type 2 lepra reactions are a rare initial presentation in leprosy that can complicate the disease with vesiculo‐bullous lesions. Early recognition and differentiation from other bullous disorders are critical for timely corticosteroid and multidrug therapy initiation to improve patient outcomes.

## Graphical abstract

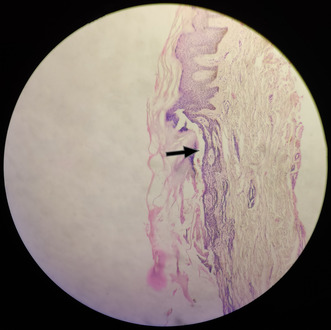

## 1 Introduction

Leprosy, also known as Hansen's disease, is an infamous chronic granulomatous infection caused by *Mycobacterium leprae* and *Mycobacterium lepromatosis*. These pathogens predominantly affect the skin and peripheral nerves. Although leprosy is known to have been eradicated in many parts of the world, it remains a major concern in developing countries, including India. Lepra reactions are immunological reactions that can complicate the course of the disease and can be a significant burden to the patient, irrespective of the status of multidrug therapy. That is, these reactions can occur before, during, or after the completion of multidrug therapy [1].

Lepra reactions can manifest in two types: type 1 and type 2 reactions. Type 2 reactions, often referred to as erythema nodosum leprosum (ENL), typically present with constitutional symptoms, accompanied by crops of painful, erythematous, cutaneous nodules, or plaques, with patients frequently experiencing multiple episodes. This type of reaction is commonly associated with lepromatous type of leprosy [2].

Type 2 lepra reaction presenting as bullous lesions is exceedingly rare and is often challenging to diagnose as it may look similar to other causes of bullous lesions. There are only very few cases reported of this unique presentation in the medical literature. This article offers a comprehensive account of this distinctive presentation, aiming to improve diagnosis and treatment of this presentation to improve patient outcomes.

## 2 Case Presentation/Examination

**FIGURE 1:**
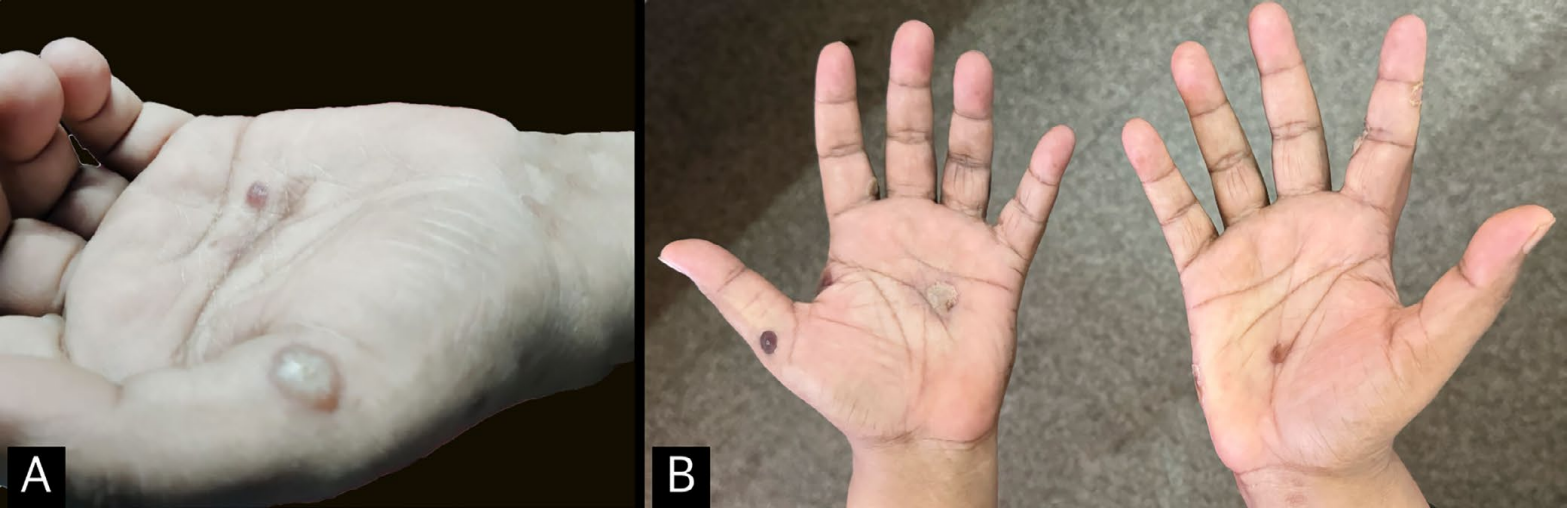
Clinical image of the findings on the palms. (A) An intact bulla seen on the lateral aspect of the left palm at the base of the thumb. (B) Image of the palm showing crusts after the rupture of bulla in Figure 1A.

**FIGURE 2:**
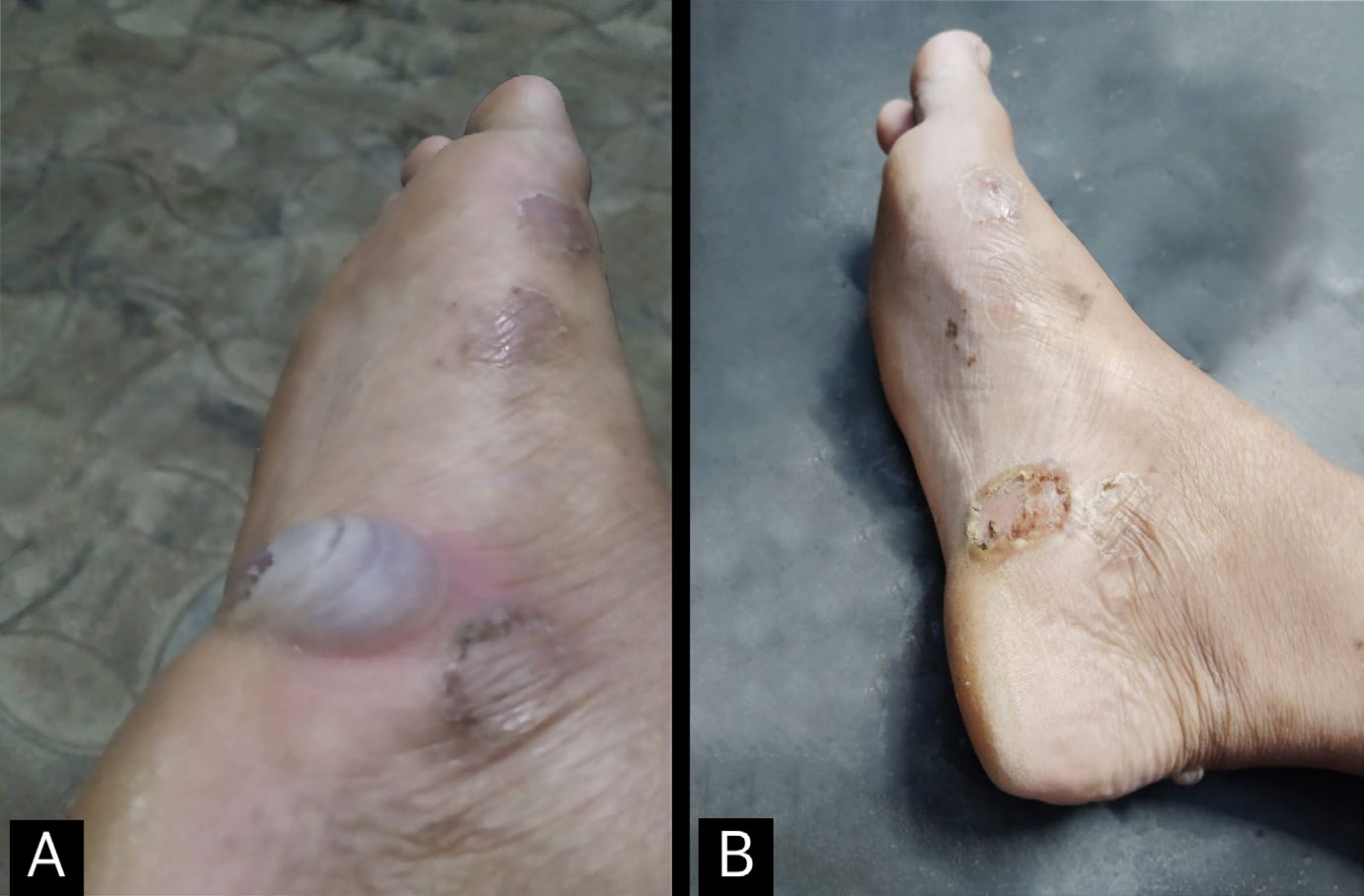
Clinical image of the findings in the sole of the right foot. (A) An intact bulla with surrounding crust from other ruptured bullae on the right sole. (B) Crust formed after the rupture of the bulla in Figure 2A.

A 42‐year‐old woman from India presented to the clinic with a 1‐year history of disseminated, clear fluid‐filled bullous lesions, primarily affecting the palms (Figure 1) and soles (Figure 2). The lesions occasionally worsen with intermittent exacerbations. The lesions were accompanied by pruritus and a burning sensation. Each episode of exacerbation was associated with constitutional symptoms, including low‐grade fever and diffuse myalgia. The patient noted that these lesions would rupture upon manipulation and then form crusts. Interestingly, these lesions had the tendency to heal spontaneously. Exacerbations have been self‐treated by the patient with oral antibiotics, topical steroids, and emollients. However, there was no history of taking drugs that could potentially trigger severe allergic reactions like Stevens–Johnson syndrome. Additionally, she had experienced paresthesias in her palms and soles for the past 2 years, with slight tremors in both upper limbs. She reported arthritis involving multiple joints. The patient did not have any relevant medical or family history. She appeared to be experiencing psychosocial stress due to the chronic nature of skin lesions and other associated symptoms.

On cutaneous examination, discrete crusted erosions and a few vesiculobullous lesions of varying sizes were noted over both the dorsal and palmar aspects of her hands. Multiple crusted superficial erosions, with few intact bullae, were noted over both her soles with a predominance over the medial aspect. Examination of the oral cavity, for lesions affecting the mucosa, revealed no abnormal findings. Nikolsky sign was negative. Ophthalmological examination and the examination of the genitals did not reveal any abnormal findings. Reduced sensation was demonstrated over both palms and soles. Also, tenderness was noted over bilateral supraclavicular nerves.

## 3 Methods (Differential Diagnosis, Investigations, and Treatment)

### 3.1 Differential Diagnosis

Based on the patient's history and physical examination, differential diagnoses included bullous pemphigoid (BP), dyshidrotic eczema, and epidermolysis bullosa acquisita (EBA), among other autoimmune and inflammatory conditions. BP was considered due to the bullous nature of the lesions but was deemed less likely given the absence of mucosal involvement and a negative Nikolsky sign. Dyshidrotic eczema was a possibility, but the presence of systemic symptoms such as fever argued against it. Also, the lack of trauma history argued against EBA. The presence of recurrent inflammation, polyarthritis, and neuropathy raised suspicion for an atypical bullous type 2 lepra reaction, or erythema nodosum leprosum (ENL), which, given the endemic context, warranted further diagnostic evaluation to confirm leprosy as an underlying cause.

### 3.2 Investigations

Routine blood investigations were conducted (Table 1). Blood glucose levels remained within normal limits. A nerve conduction study confirmed bilateral upper limb and lower limb sensorimotor axonal polyneuropathy involving the bilateral tibial, peroneal, and ulnar nerves. A Tzanck smear did not yield any significant findings.

**TABLE 1:** Routine blood tests of this patient.

Investigation	Patient value	Reference range
Total count	10,400 cells/mm^3^	4500–11,000 cells/mm^3^
Hemoglobin	12.2 g/dL	Male: 13.5–17.5 g/dL Female: 12.0–16.0 g/dL
Hematocrit	36.5%	Male: 41%–53% Female: 36%–46%
Platelet count	347,000 cells/mm^3^	150,000–400,000 cells/mm^3^
C‐reactive protein (Latex fixation test)	Negative	Negative
**Erythrocyte sedimentation rate**	**28 mm/h**	**Male: 0–15 mm/h** **Female: 0–20 mm/h**
Random blood sugar	91 mg/dL	≤ 200 mg/dL
Serum urea	14 mg/dL	7–18 mg/dL
Serum creatinine	0.7 mg/dL	0.6–1.2 mg/dL
Total bilirubin	0.4 mg/dL	0.1–1.0 mg/dL
Direct bilirubin	0.2 mg/dL	0.0–0.3 mg/dL
Indirect bilirubin	0.2 mg/dL	0.2–0.8 mg/dL
Alkaline phosphatase	98 U/L	25–100 U/L

*Note:* The erythrocyte sedimentation rate (in bold) was noted to be elevated slightly beyond the reference range.

**FIGURE 3:**
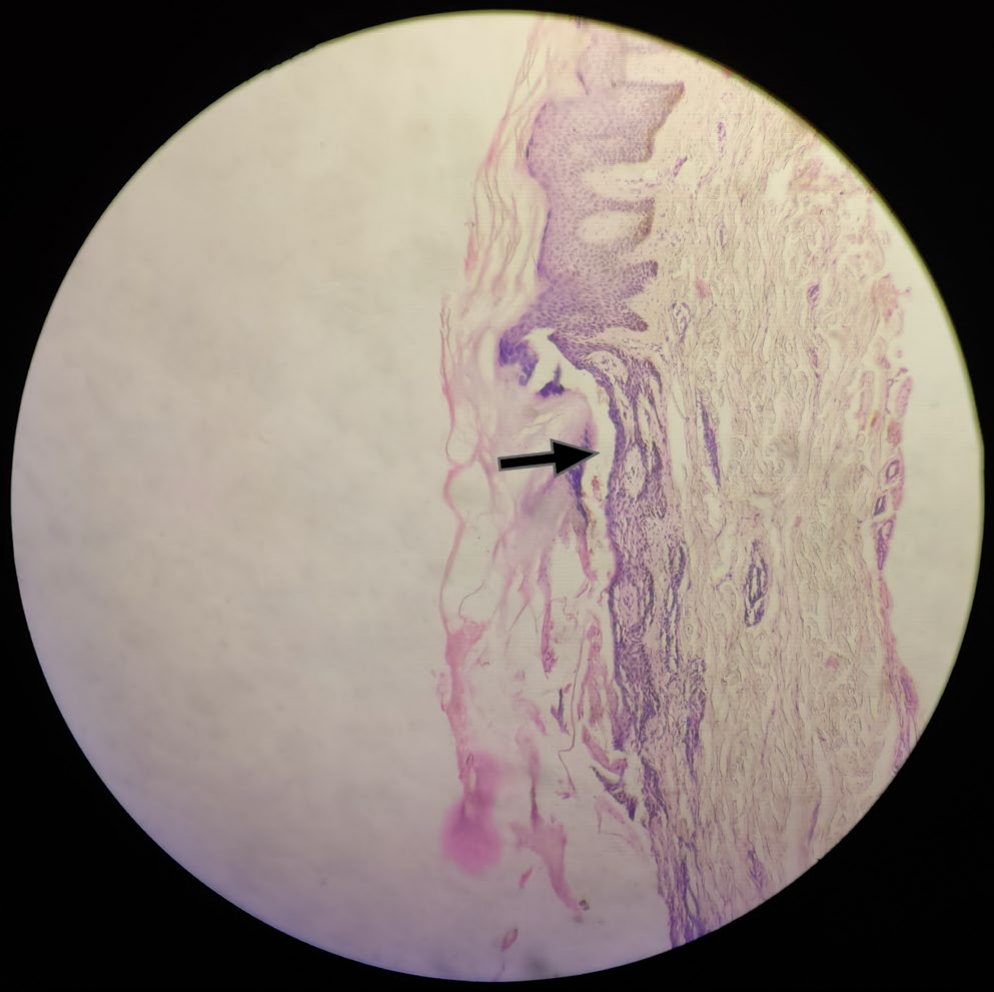
Microphotograph of the punch biopsy showing subepidermal bullous lesion marked with a black arrow (hematoxylin and eosin stain; 10× magnification).

Typical features, especially recurrent crops of lesions, raised suspicion for leprosy. Consequently, a slit skin smear from the bilateral earlobes and acid‐fast staining for the fluid from the bullae were conducted. Both tests returned positive results, indicating the presence of acid‐fast bacilli, with bacteriological indices of 3+ and 4+, respectively, suggesting multibacillary lepromatous leprosy. A 4 mm punch biopsy was taken from an intact vesicle over the left lateral malleolus and was studied under the microscope. A subepidermal bullous lesion with chronic inflammatory cell infiltrates in the underlying dermis was visualized. All these investigations aided in the diagnosis and the subsequent treatment for this patient (Figure 3).

### 3.3 Treatment

Symptomatic treatment was initiated, including the application of topical zinc ointment twice daily, tablet cetirizine 10 mg Hora Somni (for pruritus), and tablet amitriptyline 50 mg Hora Somni (for burning sensation). Proper wound care and hygiene were also suggested.

The bullous lesions were managed with a course of systemic corticosteroids, using prednisolone tablets at a dosage of 60 mg per day. Concurrently, multidrug therapy for multibacillary (lepromatous) leprosy, consisting of rifampicin 600 mg once monthly, dapsone 100 mg once daily, and clofazimine 50 mg once daily, was initiated as per the guidelines suggested in the National leprosy eradication program by the Government of India [3].

## 4 Conclusion and Results (Outcome and Follow‐Up)

**FIGURE 4:**
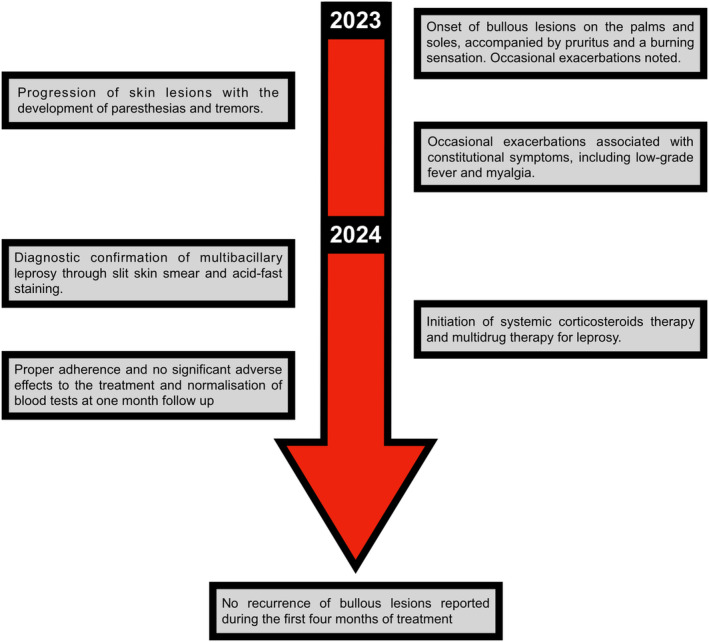
Timeline of the events in this case.

This treatment proved highly effective. The patient had adhered to the medication schedule as prescribed, and she tolerated the treatment well with no significant adverse effects. The repeat blood tests, at the 1‐month follow‐up visit, came back normal. New bullous lesions had ceased to occur, and existing lesions had begun to heal. The steroid dosage was then gradually tapered. On telephonic follow‐up after the first 4 months of treatment, the patient did not experience any recurrence of bullous lesions. The timeline of events in this case is given in Figure 4.

In this article, we provided an account of an interesting presentation of type 2 lepra reactions with bullae in a case of multibacillary lepromatous leprosy. Type 2 lepra reactions with bullae were the initial presentation in this case, and there was prompt improvement in symptoms with steroid therapy. Bullous lesions in leprosy are indeed rare, but it is crucial to consider this slight possibility when confronted with a similar clinical enigma. Ensuring an accurate diagnosis plays a pivotal role in providing the correct treatment and facilitating the prompt recovery of patients. In the ever‐evolving field of medicine, a comprehensive understanding of such unusual presentations is vital to delivering the highest standard of care to patients. Ongoing research and studies that delve into such intricacies will undoubtedly enhance our ability to diagnose, treat, and ultimately improve patient outcomes.

## 5 Discussion

Leprosy is often challenging to diagnose due to the diverse clinical presentations it can assume. Bullous lesions in leprosy further complicate the diagnostic process. An accurate diagnosis of type 2 lepra reactions in the presence of such bullous lesions is crucial because they can mimic other similar dermatological conditions like bullous pemphigoid, pemphigus vulgaris, Steven–Johnson syndrome (SJS), toxic epidermal necrolysis (TEN), bullous impetigo, Sweet's syndrome, etc. Differentiating between bullous type 2 lepra reaction lesions and other dermatological disorders is of paramount importance to provide appropriate care to the patient. The episodic appearance of the lesions, sparing of oral mucosa, along with the absence of acantholytic cells in Tzanck smear, effectively ruled out the possibility of pemphigus vulgaris and bullous pemphigoid. SJS and TEN are aberrant hypersensitivity reactions characterized by widespread blistering of skin along with the involvement of mucous membranes. They typically result from the use of certain medications. A negative history for recent consumption of such drugs and the absence of mucosal lesions were instrumental in ruling out SJS and TEN.

Lepra reactions are a consequence of the dynamic nature of host immune response against *Mycobacterium leprae* and may occur before (as in our case), during, or following the completion of multidrug therapy for leprosy. These reactions are responsible for most of the damage, including permanent nerve damage, disability, and deformity, inflicted by this disease. Type 2 lepra reactions are immune complex‐mediated reactions. Intercurrent infections, vaccination, stress, pregnancy, lactation, and puberty have been implicated as possible triggers for type 2 reactions [4]. Type 2 lepra reaction is a systemic inflammatory response characterized by neutrophil infiltration, activation of complement and extravascular immune complexes, along with high levels of tumor necrosis factor (TNF)‐α in tissue lesions and circulation. Increased expression of various cytokines, including interleukin (IL)‐4, IL‐5, IL‐6, IL‐8, and IL‐10, has been noted. Recent studies also reveal the possible role of T helper (Th)‐17 cells in the pathophysiology of type 2 lepra reactions [5, 6].

Type 2 lepra reactions affect roughly 50% of patients with lepromatous leprosy [6]. While type 2 lepra reactions typically present with tender erythematous nodules, instances of atypical presentations of type 2 lepra reactions, such as vesiculo‐bullous lesions (46% of atypical presentations), ulcero‐necrotic lesions (41%), erythema multiforme‐like lesions (28%), Sweet's syndrome‐like lesions (11%), and pustules (9%) are rarely found in the annals of medical literature [7]. Our patient exhibited a rare, atypical cutaneous presentation of type 2 lepra reactions, in the form of bullous lesions. Other features of type 2 lepra reactions include nerve function impairment, arthritis, dactylitis, iritis, osteitis, orchitis, lymphadenitis, and nephritis [8]. In our patient, extensive nerve function impairment and polyarthritis were observed. Nerve conduction study findings were suggestive of sensory and motor axonal polyneuropathy, which is a typical finding in patients with leprosy [9]. Bullous type 2 lepra reactions have limited documentation in medical literature. One of the earliest records of such an atypical presentation dates back to a case report by Gibb JA and Aberd CM in 1898 [10]. A possible hypothesis for the formation of these bullae is that they may be due to leukocytoclastic vasculitis or severe dermal edema. Bullae may also be an indicator of higher bacillary load [11].

The mainstay of treatment in a patient with moderate to severe type 2 lepra reactions includes systemic corticosteroids, namely prednisolone, while thalidomide has also proven to be an effective alternative [5, 12]. Some studies have explored the use of immunosuppressive drugs, including azathioprine and cyclosporine, with minimal beneficial results [12]. Also, TNF‐α inhibitors have been employed in some refractory cases [13]. These bullous lesions can leave permanent scars and may also become complicated by superimposed skin infections. Therefore, aside from addressing the underlying cause, supportive care for the bullous lesions through proper wound care is also essential. Once the symptoms are under control, long‐term treatment with multidrug therapy against leprosy and regular follow‐up are recommended. Leprosy, being a chronic disease, necessitates continuous monitoring to detect relapses and emerging complications to ensure the well‐being of the patient.

## Author Contributions

**Hrithik Dakssesh Putta Nagarajan:** conceptualization, data curation, formal analysis, funding acquisition, investigation, methodology, project administration, resources, software, supervision, validation, visualization, writing – original draft, writing – review and editing. **Balakrishnan Kamaraj:** project administration, resources, validation, writing – review and editing. **Keerthivasan Selvanathan:** conceptualization, data curation, formal analysis, resources, validation, writing – original draft. **Pruthvi Raj Kukunoor:** software, validation, writing – review and editing. **Katta Manichandana:** software, validation, writing – review and editing. **Katta Manideep:** validation, writing – review and editing. **Biki Kumar Sah:** validation, writing – review and editing.

## Ethics Statement

Ethical approval was not required for this case report, as it describes the clinical management of a patient and does not involve any experimental intervention. All clinical procedures followed were in accordance with institutional guidelines and the Declaration of Helsinki.

## Consent

Written informed consent was obtained from the patient for the publication of this case report and any accompanying images.

## Conflicts of Interest

The authors declare no conflicts of interest.

## Data Availability

The data that support the findings of this study are available from the first author (pnhrithik@gmail.com) and the corresponding author upon reasonable request.
